# Outcomes of All-Epiphyseal Anterior Cruciate Ligament (ACL) Reconstruction in Skeletally Immature Patients: A Systematic Review and Meta-Analysis

**DOI:** 10.7759/cureus.94359

**Published:** 2025-10-11

**Authors:** Ahmed Elnewishy, Mohamed Elgamal, Sohaib Shah, Ahmed Hamada, Mohamed A Ali, Mahmoud Noureldin, Ziad El Menawy, Kayden Chahal

**Affiliations:** 1 Trauma and Orthopaedics, Royal Berkshire Hospital, Reading, GBR; 2 Trauma and Orthopaedics, Southend Hospital, Southend-on-Sea, GBR; 3 Trauma and Orthopaedics, Maidstone and Tunbridge Wells NHS Trust, Royal Tunbridge Wells, GBR; 4 Trauma and Orthopaedics, Royal Devon and Exeter Hospital, Exeter, GBR; 5 Trauma and Orthopaedics, Royal Sussex County Hospital, Brighton, GBR; 6 Trauma and Orthopaedics, University Hospitals Sussex NHS Foundation Trust, Brighton, GBR; 7 Internal Medicine, Zayed Hospital, Abu Dhabi, ARE; 8 Trauma and Orthopaedics, National Health Service, London, GBR

**Keywords:** all-epiphyseal acl reconstruction, growth disturbance, knee joint stability, pediatric acl, skeletally immature, systematic review and meta-analysis

## Abstract

All-epiphyseal reconstruction of the anterior cruciate ligament (ACL) has gained traction as a way to stabilise the knees of children and adolescents, without violating their open physes. Nevertheless, evidence is still mixed regarding its clinical benefits and complication profile. This systematic review and meta-analysis, therefore, set out to determine the procedure’s effectiveness and safety in paediatric patients with open growth plates.

PubMed, Scopus, Web of Science, and Google Scholar were systematically searched up to July 2025 for studies reporting outcomes of all-epiphyseal ACL reconstruction in skeletally immature patients. Eligible studies reported at least one clinical or radiological outcome. Meta-analyses were performed using standardised mean differences (SMDs) and odds ratios (ORs).

Six studies (n = 150 patients) were included. Meta-analysis showed a significant improvement in Lysholm knee function scores after all-epiphyseal ACL reconstruction (SMD = -2.54; 95% CI: -2.90 to -2.17; p < 0.00001), and a marked reduction in the odds of a positive Lachman test indicating instability (OR = 1026.65; 95% CI: 142.49 to 7397.31; p < 0.00001). Most patients (up to 96%) returned to sport within 8-14 months. The risk of clinically significant growth disturbance was low (<2%), and graft failure rates ranged from 4% to 17%. Mild limb overgrowth was reported, but it is rarely clinically relevant.

All-epiphyseal ACL reconstruction provides excellent knee function and stability, with a low risk of growth disturbance in skeletally immature patients. Careful technique and long-term follow-up are recommended to minimise complications.

## Introduction and background

The incidence of anterior cruciate ligament (ACL) injuries in the paediatric population has been rising over the past decade, driven by increased participation in pivoting sports and earlier specialisation. Recent population-based studies from Finland, Norway, and the United States have reported substantial increases in ACL injuries and reconstructions in children and adolescents over 10-15 years [1-3]. A Finnish study reported an incidence of 23.3 per 100,000 person-years in individuals under age 18, with a twofold increase - most marked among girls aged 13-15. Norway noted a 40% increase in boys and a 55% increase in girls for paediatric ACL reconstructions between 2005 and 2021. Contributing factors include greater sports participation, early specialisation, improved diagnosis, and sex-related biomechanical and anatomical differences [1,3-5].

Treating ACL tears in skeletally immature patients is challenging because surgery risks physeal injury, leading to limb length discrepancy or angular deformity, whereas delaying surgery or using nonoperative care risks persistent instability, meniscal damage, and cartilage wear. Most such patients are now managed surgically, with the approach guided by skeletal age - physeal-sparing options (all-epiphyseal or iliotibial band) for younger children, and transphyseal procedures for adolescents nearing maturity [6-10]. Nonoperative treatment is rarely successful, while modern surgical techniques generally achieve good function, with a low - but nonzero - risk of growth complications [11].

Traditional techniques that cross the physes raise concern for growth disturbance due to tunnel violation, with even low absolute risks taken seriously because the consequences can be permanent [12,13]. A systematic review estimated growth disturbance at approximately 2.6%, with no major difference between transphyseal and physeal-sparing methods, although the latter showed fewer other postoperative complications. Most disturbances are mild (overgrowth or valgus), with rare clinically significant cases requiring intervention [12-14]. Imaging and biomechanical studies link risk to tunnel size, position, and fixation, and occasional overgrowth has been reported even with physeal-sparing or all-epiphyseal techniques - underscoring that the risk is low, but not zero [12,15]. Careful technique, appropriate patient selection, and long-term follow-up are therefore recommended to minimise clinically significant disturbance [16].

All-epiphyseal ACL reconstruction was developed to protect the physes by placing tunnels and graft entirely within the epiphysis, sometimes using split tibial tunnels or retrodrilling to reduce tunnel size and facilitate anatomic placement [17,18]. Reports indicate low rates of physeal violation and reduced risk of significant deformity, particularly when intraoperative 3D imaging aids precise placement, though children with small epiphyses remain at risk if tunnels encroach on the physis; preoperative planning and intraoperative imaging are advised [19,20]. Comparative reviews and series show that all-epiphyseal and other physeal-sparing approaches yield good function and stability, with minimal serious growth disturbance; overgrowth is uncommon and usually mild, and angular deformity is rare with all-epiphyseal techniques [21-23].

Overall, all-epiphyseal ACL reconstruction shows promising mid- to long-term safety and functional outcomes: high International Knee Documentation Committee (IKDC) and Lysholm scores, return to pre-injury activity, and negative stability tests when performed correctly [23-25]. Growth abnormalities are uncommon (<2%) but possible; overgrowth may occur without major consequences, and graft re-tear or contralateral ACL injury rates of 9%-15% reflect the active paediatric cohort [23,26-30]. Direct comparisons suggest similar functional results across physeal-sparing methods, with all-epiphyseal possibly showing slightly higher limb length discrepancy and re-tear risk than extraphyseal techniques, while a comparative study found no increase in major complications versus transphyseal reconstruction in this age group [27,30,31].

Objective

The objective of this systematic review and meta-analysis was to evaluate the clinical outcomes, knee stability, and complication rates of all-epiphyseal ACL reconstruction in skeletally immature patients.

## Review

Methods

Search Strategy

A comprehensive search of PubMed, Scopus, Web of Science, and Google Scholar was undertaken to locate studies on all-epiphyseal ACL reconstruction in skeletally immature patients. Literature published during the 15 years leading up to July 2025 was screened with combinations of keywords and MeSH terms - “all-epiphyseal,” “physeal-sparing,” “ACL reconstruction,” “anterior cruciate ligament,” “skeletally immature,” and “paediatric.” Reference lists of pertinent papers and reviews were also hand-checked to capture any additional eligible studies.

Inclusion and Exclusion Criteria

Studies were included if they met the following criteria: clinical design (prospective, retrospective, or comparative) evaluating all-epiphyseal ACL reconstruction in skeletally immature patients with open growth plates; reporting at least one quantitative clinical outcome, such as Lysholm score, IKDC, Tegner activity scale, KT-1000 measurements, Lachman test results, or complication rates; and publication in English in a peer-reviewed journal.

Studies were excluded if they were case reports, technical notes, review articles, or biomechanical or cadaveric studies. Additional exclusions included studies that did not involve the all-epiphyseal technique, lacked sufficient outcome data, or represented duplicate publications from the same patient cohort.

Outcome Measures

The primary outcomes assessed were postoperative knee function (Lysholm and IKDC scores), objective knee stability (Lachman test and KT-1000), and complication rates, including graft rupture and growth disturbances. Secondary outcomes included return to sport, activity level (Tegner score), and radiographic assessments of limb alignment, when reported.

Data Extraction and Quality Assessment

Two reviewers independently collected data on authorship, study design, sample characteristics, surgical details, follow-up length, and outcomes. Disagreements were settled by discussion or by a third reviewer. Study quality was gauged with the Methodological Index for Non-randomised Studies (MINORS); scores classified investigations as high, moderate, or low quality.

Statistical Analysis

Meta-analysis was carried out in Review Manager (RevMan) 5.4 (The Cochrane Collaboration, London, UK). Continuous outcomes were pooled as standardised mean differences (SMDs) with 95% confidence intervals (CIs); dichotomous outcomes were summarised as odds ratios (ORs) with 95% CIs. Fixed-effect models were used unless heterogeneity - evaluated by the Chi-square test and I² statistic - exceeded an I² threshold of 50%, in which case heterogeneity was judged moderate to high.

Results

Search and Study Selection

Following PRISMA guidelines, we interrogated PubMed, Scopus, Web of Science, and Google Scholar for studies on all-epiphyseal ACL reconstruction in skeletally immature patients, covering publications through July 2025. Search strings combined terms such as “all-epiphyseal,” “physeal-sparing,” “ACL reconstruction,” “skeletally immature,” and “paediatric anterior cruciate ligament.” Reference lists of relevant articles and reviews were also hand-searched for additional records.

**Figure 1:**
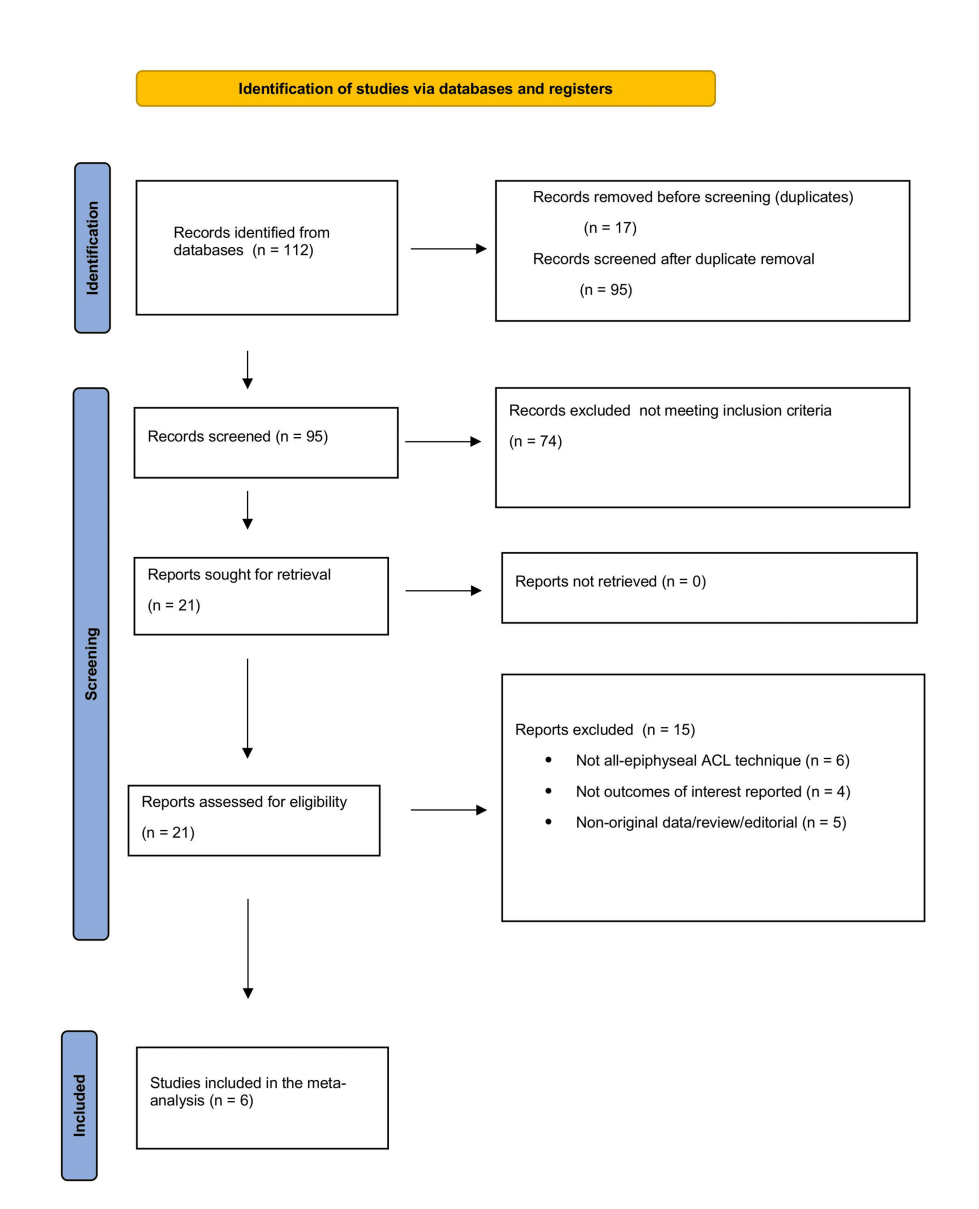
PRISMA flow chart for the included studies.

The search produced 112 citations. After eliminating 17 duplicates, 95 records advanced to title-and-abstract screening; 74 of these were excluded for not satisfying the inclusion criteria - namely, original prospective, retrospective, or comparative studies reporting at least one postoperative outcome of all-epiphyseal ACL reconstruction in patients with open physes. Next, 21 articles underwent full-text review. Of these, 15 were removed because they employed non-all-epiphyseal techniques, lacked the required outcome data, or represented non-original literature (e.g., reviews and editorials). Ultimately, six studies met every eligibility benchmark and were incorporated into the quantitative synthesis. The selection process is summarised in the PRISMA flow diagram (Figure 1).

Study Characteristics

A total of six studies, with 150 patients, were included in the final quantitative synthesis, comprising prospective and retrospective case series and one comparative cohort study. The studies were published between 2010 and 2025 and were conducted across multiple countries. Sample sizes ranged from 3 to 74 skeletally immature patients, all confirmed to have open physes at the time of ACL reconstruction. The mean or median patient age across studies ranged from approximately 10 to 13 years, with both males and females represented.

All included studies evaluated outcomes following all-epiphyseal ACL reconstruction, most commonly using physeal-sparing techniques with autologous hamstring grafts. Follow-up durations varied from 12 months to over 4 years. Reported outcome measures included validated clinical scores (Lysholm, IKDC, Knee Injury and Osteoarthritis Outcome Score (KOOS), and Tegner), objective stability testing (Lachman, KT-1000, and pivot shift), return to sport, complications (including graft failure and growth disturbances), and radiographic assessments of limb length and axis [29].

Study designs were predominantly non-comparative (Level IV evidence), except for one comparative cohort study. The majority of studies provided either prospectively or retrospectively collected outcome data, with varying degrees of methodological rigour. A summary of key study characteristics is shown in Table 1.

**Table 1:** Summary of study characteristics, including sample size, patient demographics, intervention details, follow-up duration, complications, and primary outcomes, for studies evaluating all-epiphyseal ACL reconstruction in skeletally immature patients. Lysholm: Lysholm Knee Scoring Scale; IKDC: International Knee Documentation Committee; KOOS: Knee Injury and Osteoarthritis Outcome Score; KT-1000: Knee Laxity Assessment; ROM: Range of Motion; ACL, Anterior Cruciate Ligament; ADL: Activities of Daily Living; QOL: Quality of Life; DB-ACL: Double-Bundle Anterior Cruciate Ligament; FTA: Femorotibial Angle; HSS Pedi-FABS: Hospital for Special Surgery Pediatric Functional Activity Brief Scale; MARX: Marx Activity Rating Scale; RIS: Resorbable Interference Screws

Study	Study Design	Sample Size	Level of Evidence	Patient Demographics	Intervention Details	Follow-Up Duration	Outcome Measures	Results	Complications	Conclusions
Cordasco et al. [24]	Prospective case series	23 skeletally immature athletes	IV	17 males (mean age 12.6), 6 females (mean age 11.3); mean age overall 12.2 yrs (range 9.9-14.5); all with 3-6 years growth remaining; sports: soccer, skiing, lacrosse, etc.	All-inside, all-epiphyseal ACL reconstruction with hamstring autograft (sockets created all-epiphyseal with adjustable-loop suspensory fixation); standardised rehab protocol.	Minimum 2 years (mean 32.1 months, range 24-45)	IKDC, Lysholm, Marx Activity, HSS Pedi-FABS, Lachman, pivot shift, KT-1000, radiographs, MRI, return to sport, second surgery incidence.	IKDC: 94.6 ± 4.9; Lysholm: 97.9 ± 4.0; Marx: 13.4 ± 3.6; HSS Pedi-FABS: 23.9 ± 7.0; Lachman and pivot shift negative in all; KT-1000 side-to-side diff: 0.9 ± 0.5 mm; 22/23 (96%) returned to unrestricted sport at a mean 13.5 months (range 8-22).	2 required second surgery (8.7%): 1 ipsilateral rerupture (treated with revision), 1 late medial meniscal repair failure; 6 had leg length discrepancy >5 mm (all femoral overgrowth, range 6-18 mm); 2 had >15 mm; No clinically significant angular deformity, growth arrest, or symptomatic discrepancy.	All-inside, all-epiphyseal ACL reconstruction yields excellent functional outcomes, high return-to-sport rate, low rerupture/reoperation rates, and very low risk of clinically significant growth disturbance or angular deformity in skeletally immature athletes.
Knörr et al. [32]	Prospective cohort (case series)	74 patients	IV	Mean age: 11.7 ± 1.3 yrs (boys: 11.9 ± 1.3, girls: 10.7 ± 1.3); 60 males (81.1%), 14 females (18.9%); Right knee: 56.8%; Prepubertal (Tanner I-III).	Arthroscopic all-epiphyseal ACL reconstruction using semitendinosus-gracilis tendon autograft, fixed with intra-epiphyseal RIS; minimal notchplasty in 90.5%.	Mean: 4.1 ± 1.5 years (range 2-7)	Clinical laxity (Lachman, pivot shift, Lerat method), Lysholm score, Tegner score, radiographic limb length/axis, MRI, and patient satisfaction.	Side-to-side laxity improved from 7.6 ± 1.4 mm pre-op to 2.8 ± 2.1 mm post-op (p < 0.01); 91.9% had excellent/good Lysholm scores (mean 94.5 ± 11.4), improved from 60.9 ± 14.7; Tegner score: 5.4 both pre-injury and post-op; Mean time to return to sport: 8 months; No relevant axial deformity or limb length discrepancy; mean overgrowth: 6.5 mm.	17 cases (23%): Graft failure: 3 (4.1%); Meniscal tears: 20 (27%); New tears: 4 (5.4%); Meniscal repair failure: 2/16 (12.5%); Screw-related incident: 7 (9.5%); Septic arthritis: 1 (1.4%); No clinically significant growth disturbances.	All-epiphyseal ACL reconstruction in skeletally immature patients provides excellent knee function, low rerupture/complication rates, and preserves growth/limb axis. Recommended as a safe, effective procedure in this population.
Koch et al. [33]	Retrospective case series	12 patients (13 knees)	IV	Median age: 12.1 yrs (range 10.4-13.4); 10 boys, 2 girls; all prepubescent with open physes.	All-epiphyseal ACL reconstruction (physeal-sparing, Anderson technique), autologous semitendinosus/gracilis grafts, femoral Flipptack fixation, tibial screw (no interference screws).	Median 54 months (range 39-80)	IKDC-2000 (objective and subjective), Lysholm, Tegner, KT-1000, radiographs (limb length, alignment).	IKDC: 5 A, 6 B, 2 C; Median subjective IKDC: 88.5 (74.7-98.9); Lysholm median: 93 (73-100); Tegner: pre-injury 7.7, follow-up 6.2; KT-1000 mean side-to-side diff: 1.5 ± 2.5 mm (5 knees >3 mm); No extension deficit, 4 knees 5-10° less flexion.	2 graft failures (reoperation), 2 significant leg overgrowth (>10 mm; 1 had +21 mm and varus, 1 had +16 mm), 4 minor overgrowth (5-10 mm), 1 late meniscal tear needing suture, no growth arrest.	Acceptable clinical/functional outcomes, but high rate of limb overgrowth and complications. Regular follow-up is recommended until growth completion. Failure/reoperation rates are higher than in adults.
Lawrence et al. [34]	Prospective case series (case report/technical note)	3 patients	IV	3 boys, ages 10-12, Tanner Stage 1, prepubescent, all with open physes, ACL tear (2 also with meniscal tear).	All-epiphyseal ACL reconstruction using intraoperative CT-guided anatomic tunnels; graft: autologous hamstrings (2 cases), allograft tibialis posterior (1 case); fixation: retrograde interference screw in tibia and interference screw in femur; meniscal repair as needed.	Minimum 1 year	Lachman, pivot shift, KT-1000, ROM, strength, growth disturbance, return to sports.	All had stable knees (Lachman, KT-1000 ≤1 mm diff), full ROM, symmetric flex/ext strength, return to sports with ACL brace; no growth disturbance or leg length discrepancy at follow-up.	None reported	Anatomic all-epiphyseal ACL reconstruction with 3D imaging minimises the risk of growth disturbance, restores stability and function in skeletally immature patients; the technique is feasible and safe in the short term. Longer-term outcomes needed.
Roberti di Sarsina et al. [35]	Retrospective case series	20 patients	IV	Age at surgery: mean 12.3 (1.7) yrs (range 8-13); 10 males, 10 females; Tanner I-IV; regular sport participation; open proximal tibial physis.	All-epiphyseal single-bundle “over-the-top” ACL reconstruction + extra-articular lateral tenodesis using hamstring tendons.	Mean 54 months (range 34-123)	Lysholm, KOOS, Tegner (pre-injury, pre-op, post-op), IKDC (post-op), KT-1000, Rolimeter, pivot shift, limb length discrepancy, axial alignment (radiographs).	Lysholm: improved from 40 (22; 65) pre-op to 100 (95; 100) post-op; KOOS: 59 (42; 73) to 99 (97; 100); Tegner: 2 (2; 2) pre-op to 7 (3; 9) post-op; pre-injury 8 (7; 9); IKDC: 19 A, 1 B; KT-1000: median side-to-side difference 0.0 mm (-0.4; 1.0) (standard force); All returned to sport, with 60% at the same pre-injury activity level.	3 minor limb length/axis deviations: (1) 0.6 cm lengthening + 4° varus, (2) 1 cm lengthening, (3) 3° varus (vs. Contralateral); No graft failures, no major growth disturbances.	All-epiphyseal “over-the-top” ACL reconstruction with lateral tenodesis is safe and effective in skeletally immature athletes, providing excellent function, low failure, and minimal growth/axis disturbance.
Sasaki et al. [36]	Retrospective cohort study (prospectively collected data)	18 all-epiphyseal DB-ACL reconstructions (control: 84 conventional DB-ACL)	III	All-epiphyseal group: Mean age 12.4 ± 1.2 yrs, 10 females, 8 males; all with open physes.	All-epiphyseal double-bundle ACL reconstruction with hamstring tendon autograft; both femoral and tibial tunnels placed entirely in the epiphysis, avoiding physis; standardised rehab protocol.	Mean 41.6 ± 20.1 months (min 24)	KT-1000 side-to-side difference, Lachman/pivot shift tests, KOOS, radiographic femorotibial angle, isokinetic strength, second ACL injury (graft rupture/contralateral).	KT-1000 improved from 6.1 ± 2.4 to 0.6 ± 0.9 mm (p = 0.001); KOOS (Pain 97, Symptoms 93.5, ADL 99.6, Sport 97.3, QOL 92.6); 20% Lachman grade 1, 22% pivot shift grade 1 at 24 mo; 26.7% (4/15) had angular deformity; ipsilateral graft rupture: 16.7% (3/18); contralateral ACL tear: 11.1% (2/18).	26.7% (4/15) radiographic angular deformity (>3° FTA); 16.7% ipsilateral graft rupture; 11.1% contralateral ACL injury; no symptomatic leg length discrepancy; no additional surgery for deformity.	All-epiphyseal DB-ACL reconstruction achieved satisfactory subjective and objective outcomes, but with higher rates of residual laxity and angular deformity versus conventional techniques. Ipsilateral graft rupture rate is relatively high. Growth disturbances (angular deformity) remain a concern.

Quality Assessment of Included Studies

The methodological soundness of the six eligible studies was appraised with the MINORS, a validated checklist for estimating bias in non-randomised surgical research. MINORS comprises 12 items that evaluate critical design elements: a clear statement of study objectives, consecutive patient inclusion, data-collection procedures, suitability of endpoints, outcome assessment, adequacy of follow-up, and, when applicable, comparability between groups, alongside statistical analysis. Each element is graded 0 (not reported), 1 (reported but inadequate), or 2 (reported and adequate). Consequently, non-comparative investigations can attain a maximum of 16 points, whereas comparative studies may score up to 24.

**Figure 2:**
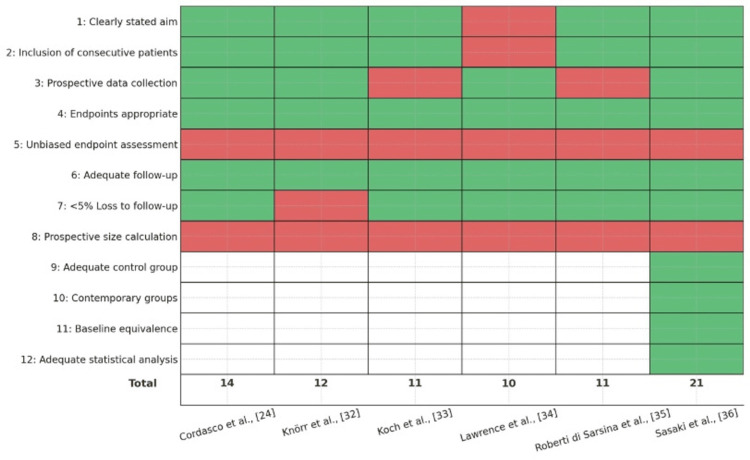
Methodological quality assessment of included studies using the MINORS tool. Green denotes criterion met; red denotes criterion not met; white denotes criterion not applicable to that study. MINORS: Methodological Index for Non-randomised Studies

The majority of studies achieved moderate methodological quality, with most scoring well for clarity of study aim, use of validated outcome measures, and adequacy of follow-up. However, lower scores were frequently observed for unbiased assessment of outcomes (e.g., blinding), and none of the studies reported a prospective sample size calculation. Only one study was comparative and scored highly across additional comparative domains [36], including group comparability and appropriate statistical analyses. A summary of MINORS scores, by item and study, is illustrated in Figure 2.

Each study was evaluated across 12 domains; green indicates the criterion was adequately reported (score 2), red indicates it was inadequately reported or not reported (score 1 or 0), and white indicates the item was not applicable (NA) for single-arm studies. Total scores are shown at the bottom (maximum 16 for non-comparative studies and 24 for comparative studies). Higher scores reflect stronger methodological quality.

Results of meta-analysis

Knee Function Recovery (Lysholm Score)

**Figure 3:**
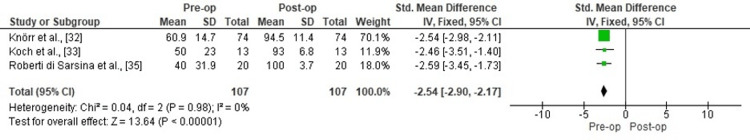
Forest plot showing pre- and post-operative Lysholm score improvement after all-epiphyseal ACL reconstruction. ACL: Anterior Cruciate Ligament

The meta-analysis of knee function recovery demonstrated a statistically significant improvement in Lysholm scores following all-epiphyseal ACL reconstruction (SMD = -2.54, 95% CI: -2.90 to -2.17, p < 0.00001). No heterogeneity was observed among the included studies (Chi-square = 0.04, df = 2, p = 0.98; I² = 0%). Figure 3 illustrates the forest plot for this outcome.

Anterior Knee Stability (Lachman Test)

**Figure 4:**
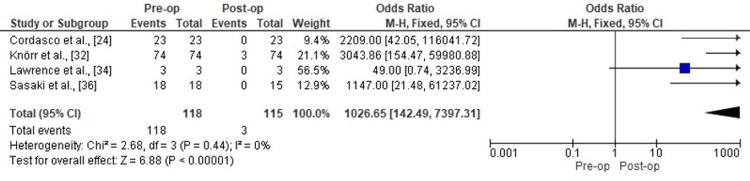
Forest plot comparing pre- and post-operative Lachman test positivity following all-epiphyseal ACL reconstruction. ACL: Anterior Cruciate Ligament

Meta-analysis of the Lachman test revealed a marked reduction in the odds of a positive (unstable) test result postoperatively (OR = 1026.65, 95% CI: 142.49 to 7397.31, p < 0.00001). Heterogeneity was low (Chi-square = 2.68, df = 3, p = 0.44; I² = 0%). Figure 4 shows the forest plot for this outcome.

Discussion

Management of ACL injuries in skeletally immature patients continues to evolve, with the all-epiphyseal ACL reconstruction technique now widely regarded as a leading option for restoring stability while minimising the risk of growth disturbance. Most of the available literature supports this approach, but there remain areas of debate and some notable complications.

Numerous prospective and retrospective studies have demonstrated excellent functional outcomes after all-epiphyseal ACL reconstruction. For example, Cordasco et al. [24] prospectively evaluated skeletally immature athletes who underwent all-inside, all-epiphyseal ACL reconstruction and reported mean IKDC and Lysholm scores above 90 at two years postoperatively, with nearly all patients achieving negative Lachman and pivot-shift tests. Notably, 96% returned to their previous level of sport, indicating strong functional recovery and high patient satisfaction. Although six athletes developed leg length discrepancies greater than 5 mm, only two had discrepancies exceeding 15 mm, and no symptomatic angular deformity was observed, supporting the general safety of the technique.

Wall et al. [23] similarly followed a cohort of 27 patients and observed excellent mean IKDC scores (mean 94), minimal clinically relevant growth disturbances, and satisfactory stability over almost four years of follow-up. They did report, however, a graft failure rate of 15% and a number of secondary meniscal procedures, illustrating that, while outcomes are generally excellent, re-injury is not uncommon in active paediatric populations.

These positive findings have been corroborated by Pennock et al. [29], who described a modified all-epiphyseal technique and found high rates of return to sport (94%), strong Lysholm scores (mean 93), and very few cases of limb length discrepancy or angular deformity at mid-term follow-up.

However, not all evidence is uniformly supportive, and there are important considerations regarding complication profiles. Salvato et al. [20] highlighted that children with a low distal femoral epiphysis height may face an increased risk of clinically significant growth disturbance, including growth arrest and limb length discrepancy, even with a technically perfect all-epiphyseal reconstruction. This underlines the need for careful preoperative imaging and patient selection, especially in smaller or younger children.

When comparing all-epiphyseal with other physeal-sparing techniques, Zakharia et al. [27] performed a systematic review and found that both all-epiphyseal and Micheli-Kocher (MK) techniques provided high rates of return to sport and excellent functional outcomes, but the all-epiphyseal group had slightly higher rates of growth disturbance (1.5%), angular deformity (1.3%), and graft failure (10.6%) than the MK group. However, these absolute differences were small, and both approaches remain viable options.

A meta-analysis by Knapik and Voos [30] suggested that all-epiphyseal reconstruction is associated with higher rates of limb length discrepancy and rerupture, as well as a slightly lower return-to-activity rate when compared to extraphyseal techniques. These findings indicate that, while the all-epiphyseal approach is generally effective, patients and families should be counselled about the small but real risks of certain complications, especially in highly active children.

On the other hand, large retrospective studies comparing all-epiphyseal and transphyseal ACL reconstruction have not demonstrated significant differences in overall complication rates, including graft rupture, contralateral ACL injury, or new meniscus tears. Patel et al. [31] found that, after controlling for confounders, complication risks were comparable between the two methods, thus supporting the all-epiphyseal technique as a safe physeal-sparing option for growing children.

Finally, patient-reported outcomes after all-epiphyseal ACL reconstruction remain high, even among those who experienced rerupture. Ranade et al. showed that rerupture led to lower functional scores, but the overall cohort still reported high satisfaction and return to activity, reinforcing the durability and value of this surgical approach in the paediatric population [36].

Limitations

The main limitations of this review are the reliance on mostly retrospective case series with small sample sizes and short follow-up periods. Variation in surgical techniques and inconsistent reporting of complications also make it difficult to directly compare outcomes. Additionally, limited long-term data may underestimate the true rate of growth disturbances or late complications.

## Conclusions

All-epiphyseal ACL reconstruction is an effective surgical technique for skeletally immature patients, providing strong knee stability, excellent functional outcomes, and a low risk of clinically significant growth disturbance when performed with care. Most patients can expect a successful return to their pre-injury level of activity and high levels of satisfaction. Nevertheless, there remains a small risk of limb length discrepancy, overgrowth, or graft failure - particularly among younger or highly active children. Careful surgical planning, individualised patient selection, and diligent long-term follow-up are crucial for optimising results and minimising risks. As the field evolves, ongoing research and long-term studies will be essential to refine surgical strategies and further clarify the best approach for this unique patient population.
